# INSPECT-SR: a tool for assessing trustworthiness of randomised controlled trials

**DOI:** 10.1101/2025.09.03.25334905

**Published:** 2025-09-11

**Authors:** Jack Wilkinson, Calvin Heal, Ella Flemyng, Georgios A. Antoniou, Tony Aburrow, Zarko Alfirevic, Alison Avenell, Virginia Barbour, Vincenzo Berghella, Dorothy V. M. Bishop, Esmée M Bordewijk, Nicholas J. L. Brown, Jana Christopher, Mike Clarke, Darren Dahly, Jane Dennis, Patrick Dicker, Jo Dumville, Helen Frankish, Andrew Grey, Steph Grohmann, Lyle C. Gurrin, Jill A. Hayden, James A.J. Heathers, Kylie E Hunter, Ian Hussey, Lukas Jung, Emily Lam, Toby J. Lasserson, Sarah Lensen, Tianjing Li, Wentao Li, Jianping Liu, Elizabeth Loder, Andreas Lundh, Gideon Meyerowitz-Katz, Ben W. Mol, Florian Naudet, Anna Noel-Storr, Neil E. O’Connell, Lisa Parker, Rita F. Redberg, Barbara K. Redman, Rachel Richardson, Anna Lene Seidler, Kyle Sheldrick, Emma Sydenham, Madelon van Wely, Colby J. Vorland, Rui Wang, Stephanie Weibel, Matthias Wjst, Lisa Bero, Jamie J. Kirkham

**Affiliations:** 1. Centre for Biostatistics, Manchester Academic Health Science Centre, Faculty of Biology, Medicine, and Health, University of Manchester, Manchester, UK.; 2. Cochrane Central Executive, London, UK.; 3. Manchester Vascular Centre, Manchester University NHS Foundation Trust, Manchester, UK.; 4. Division of Cardiovascular Sciences, School of Medical Sciences, Manchester Academic Health Science Centre, The University of Manchester, Manchester, UK.; 5. University of Liverpool, UK.; 6. Aberdeen Centre for Evaluation, University of Aberdeen, UK.; 7. Medical Journal of Australia and Faculty of Health, Queensland University of Technology, Brisbane, Australia.; 8. Division of Maternal-Fetal Medicine, Department of Obstetrics and Gynecology, Thomas Jefferson University, Philadelphia, USA.; 9. Department of Experimental Psychology, University of Oxford, UK.; 10. Department of Obstetrics and Gynaecology, Centre for Reproductive Medicine, Amsterdam UMC, University of Amsterdam, Amsterdam, The Netherlands.; 11. Department of Psychology, Linnaeus University, Sweden.; 12. FEBS Press, Heidelberg, Germany.; 13. Centre for Public Health, Queen’s University Belfast, Belfast, UK; 14. HRB Clinical Research Facility, School of Public Health, University College Cork, Cork, Ireland.; 15. University of Bristol, UK.; 16. Department of Epidemiology and Public Health, Royal College of Surgeons in Ireland, Ireland.; 17. School of Health Sciences, University of Manchester, Manchester, UK.; 18. The Lancet, London, UK.; 19. University of Auckland, Auckland, New Zealand.; 20. Research Integrity Editor, Cochrane, London, UK.; 21. School of Population and Global Health, The University of Melbourne.; 22. Department of Community Health & Epidemiology, Dalhousie University, Halifax, Nova Scotia, Canada.; 23. Novia Scotia Health, Halifax, Nova Scotia, Canada.; 24. Medical Evidence Project, Center for Scientific Integrity, NY, USA.; 25. NHMRC Clinical Trials Centre, University of Sydney, Australia.; 26. Institute of Psychology, University of Bern, Switzerland.; 27. Independent researcher, Heidelberg, Germany.; 28. Independent Lay Member, Unaffiliated, Cheshire, UK.; 29. Department of Obstetrics, Gynaecology and Newborn Health, Royal Women’s Hospital, University of Melbourne, Australia; 30. Department of Ophthalmology, University of Colorado Anschutz Medical Campus, USA.; 31. National Perinatal Epidemiology and Statistics Unit, Centre for Big Data Research in Health and School of Clinical Medicine, The University of New South Wales, Sydney, Australia.; 32. Centre for Evidence-Based Chinese Medicine, Beijing University of Chinese Medicine, Beijing, China.; 33. The BMJ, UK.; 34. Harvard Medical School, Boston, MA, USA.; 35. Cochrane Denmark & Centre for Evidence-Based Medicine Odense (CEBMO), Department of Clinical Research, University of Southern Denmark, Odense, Denmark.; 36. Open Patient data Explorative Network (OPEN), Odense University Hospital, Odense, Denmark.; 37. Department of Respiratory Medicine and Infectious Diseases, Copenhagen University Hospital-Bispebjerg and Frederiksberg, Copenhagen, Denmark.; 38. University of Woolongong, NSW, Australia.; 39. Department of Obstetrics Gynaecology, Monash University, Melbourne, Australia.; 40. Univ Rennes, CHU Rennes, Inserm, EHESP, Irset (Institut de recherche en santé, environnement et travail) - UMR_S, Rennes, France.; 41. Institut Universitaire de France (IUF), Paris, France.; 42. Department of Health Sciences, Centre for Health and Wellbeing Across the Lifecourse, Brunel University of London, United Kingdom.; 43. Charles Perkins Centre, Faculty of Medicine and Health, University of Sydney, Australia.; 44. Department of Medicine, UCSF Philip Lee Institute for Health Policy Studies, USA.; 45. New York University Grossman School of Medicine, New York, USA.; 46. Department of Child and Adolescent Psychiatry, University Medical Centre Rostock, Rostock, Germany; German Center for Child and Adolescent Health (DZKJ), partner site Greifswald/Rostock, Rostock, Germany.; 47. Spine Labs, Faculty of Clinical Medicine, University of New South Wales.; 48. Cochrane Central Editorial Service, London, UK.; 49. Centre for Reproductive Medicine, Amsterdam Reproduction & Development Research Institute, Amsterdam UMC, Amsterdam, The Netherlands.; 50. Department of Epidemiology and Biostatistics, Indiana University School of Public Health-Bloomington, USA.; 51. University Hospital Würzburg, Department of Anaesthesiology, Intensive Care, Emergency and Pain Medicine, Würzburg, Germany.; 52. Institut für KI und Informatik in der Medizin, Lehrstuhl für Medizinische Informatik, Klinikum rechts der Isar, Grillparzerstr. 18, D-81675 München, Germany.; 53. Center for Bioethics, and Humanities, University of Colorado Anschutz Medical Campus, Aurora, CO, USA.

## Abstract

The integrity of evidence synthesis is threatened by problematic randomised controlled trials (RCTs). These are RCTs where there are serious concerns about the trustworthiness of the data or findings. This could be due to research misconduct, including fraud, or due to honest critical errors. If these RCTs are not detected, they may be inadvertently included in systematic reviews and guidelines, potentially distorting their results. To address this problem, the INSPECT-SR (INveStigating ProblEmatic Clinical Trials in Systematic Reviews) tool has been developed to assess the trustworthiness of RCTs. This will allow problematic RCTs to be identified and excluded from systematic reviews. This paper describes the development of INSPECT-SR. The tool and an associated guidance document are presented.

## Introduction

In systematic reviews of randomised controlled trials (RCTs) the established steps are to identify all eligible trials, to appraise them using risk of bias tools, and to synthesise the results to reach a conclusion about the effectiveness and harms of an intervention. This paradigm is threatened by the presence of *problematic studies* in the literature. As defined by Cochrane, these are studies “where there are serious questions about the trustworthiness of the data or findings” (1). A study could be problematic due to research misconduct (including plagiarism, falsification, and fabrication of data or methods) or due to critical errors in study conduct that would not be flagged by risk of bias tools. If these studies are not identified as untrustworthy, they may inadvertently be included in evidence synthesis, potentially leading to treatment recommendations that are misleading or even actively harmful (2–4). Because many of these studies are not easily identifiable, it is difficult to assess the prevalence of problematic RCTs, but the available evidence is not encouraging. Carlisle estimated that 26% of RCTs submitted to the journal Anaesthesia were problematic (5). A recent review identified 847 systematic reviews that included one or more retracted RCTs in meta-analysis, and found that excluding these studies changed the size of effect in 16% of meta-analyses, and the direction of effect in just over 8% (6). It may take years for a problematic trial to be recognised and retracted, if it is retracted at all (7, 8), so these figures are likely to underestimate the problem. These delays also mean that systematic reviewers cannot rely on retraction status alone to identify problematic trials.

Risk of bias tools are not designed to identify problems of this nature, nor do they appear to be successful in doing so (9, 10). Bespoke tools for *trustworthiness assessment* are therefore needed. A variety of trustworthiness tools have recently been proposed (11–15), although they differ in terms of their development, content and structure, and the appropriateness of some of the trustworthiness checks that have been included in some of these tools is unclear (16). Given the number of problematic studies that are now being identified, and the growing number of proposed methods for detecting these studies, it was timely to assess and reach international consensus on a set of checks and principles for trustworthiness assessment.

In this article, we present the INSPECT-SR (INveStigating ProblEmatic Clinical Trials in Systematic Reviews) tool. INSPECT-SR implements a set of trustworthiness checks, selected on the basis of empirical evidence, expert consensus methods, and theoretical considerations in the form of a practical tool that will help systematic reviewers and users of primary research to assess whether an RCT is trustworthy.

## Scope of the INSPECT-SR tool

INSPECT-SR has been created to assess trustworthiness of RCTs in order to identify problematic studies. This involves checking various aspects of the trial publication and associated documents in order to reach a conclusion about the veracity of the study’s reported methods and results. The tool does not assess risk of bias, generalisability, or conflicts of interest. The tool does not require that individual participant data (IPD) are available. A tool for assessing trustworthiness using IPD has been developed (15) and INSPECT-IPD, an extension to INSPECT-SR that can be used when IPD are available, is in development (funder ref: NIHR303741). INSPECT-SR can be used on all RCTs, regardless of the area of healthcare. The tool has not been designed as a diagnostic test for fraud, since honest error that is fatal to an RCT’s conclusions cannot generally be ruled out, and it should not be deployed or interpreted as such. Concerns about a trial’s trustworthiness do not constitute an accusation of research misconduct.

## Development of the tool

**Figure 1:**
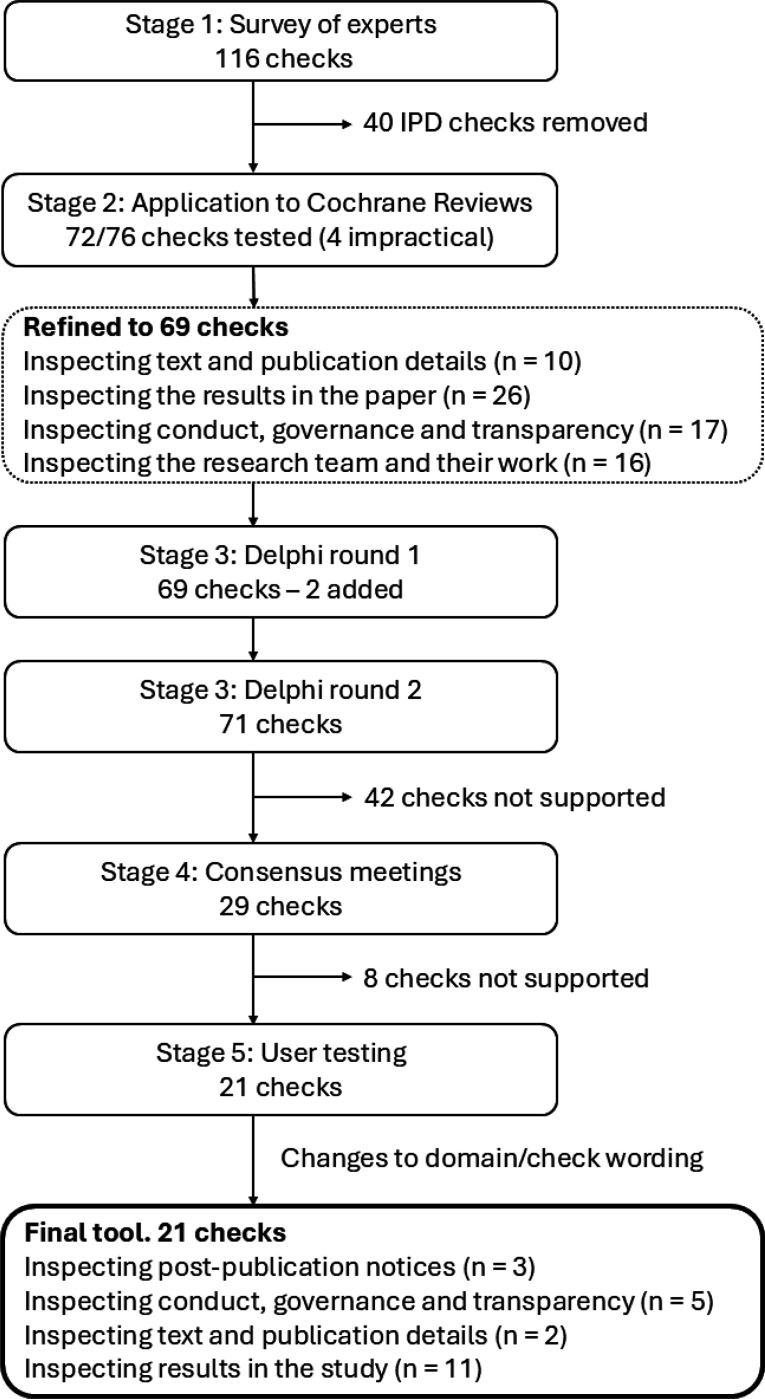
Summary of the development of INSPECT-SR

The development process for the tool has previously been described (17) and included five stages: 1) creation of a comprehensive list of trustworthiness checks using existing literature and a survey of experts, to be evaluated in subsequent stages; 2) application of the checks to 50 Cochrane Reviews to evaluate their feasibility and impact; 3) a Delphi survey to establish which of the checks are supported by expert consensus; 4) a series of online consensus meetings to determine which checks to include in the draft INSPECT-SR tool, and how they should be operationalised; and 5) user testing of the draft tool, with feedback, captured using an online survey and user workshop, to finalise the tool and accompanying guidance document. Stages 1 and 2 have been reported previously (9, 18). Here we report Stages 3 to 5 and present the final tool. Figure 1 shows the number of checks included at each stage of development.

### Candidate item list generation

A comprehensive list containing 76 trustworthiness checks was produced using existing evidence (a scoping review (19) and qualitative study (20)) and a new survey of experts (18). The list was refined in consultation with the project expert advisory panel using results from an application of the checks to RCTs in Cochrane Reviews (9). The refined list of 69 checks and associated explanations, arranged in four domains (Figure 1), can be viewed at https://osf.io/esxu7.

### Recruitment of Delphi participants

Individuals with expertise or experience in assessing potentially problematic studies and potential users of the tool were eligible to participate. Individuals were identified via professional networks of the study team and expert advisory panel, from individuals who had contacted the study team expressing interest, from authors of relevant publications, and via advertisement on social media and academic presentations. Participants were invited by personalised email, containing a unique participation link. Consent was obtained within the survey. Eligible individuals were invited to participate in Round 2 even if they had not participated in Round 1.

### Delphi survey

The survey was implemented in Qualtrics (Provo, UT) and can be viewed at https://osf.io/9nu23. Participants were asked to score each check 1 to 9 on two scales, relating to usefulness and feasibility. Usefulness was explained with the text “Do you think performing this check would help to identify problematic studies?” Feasibility was explained with the text “Do you think performing this check would be easy for a competent review team to perform when assessing trustworthiness of a study, in terms of required skill, expertise, resources, or time?” A “don’t know” option was also available. Free text boxes were included for comments, or for suggestions for additional checks, which were then included in the Round 2 survey. At Round 2, participants were presented with their own scores from Round 1, and a summary of scores from all participants. Participants were then asked to score each check again in light of the Round 1 scores. A prespecified consensus threshold was applied. Any check that scored 7 or more for usefulness by at least 80% of participants at Round 2 was considered to be supported.

The Delphi was conducted between November 2023 and April 2024. 323 and 333 individuals were invited to Rounds 1 and 2, respectively, with 148 and 134 participants completing each round. Supplementary Table 1 shows characteristics of participants. Twenty-nine checks met the consensus criterion and were then entered into the Stage 4 consensus meetings (Supplementary Table 2).

### Consensus meetings

For each of four domains, a consensus meeting was held in duplicate (eight total meetings) to accommodate participants in different time zones. Different participants were invited to meetings relating to different domains, to ensure that checks were discussed by people with relevant expertise. Results from Stages 2 and 3 were presented to participants (slides can be viewed at https://osf.io/5k7yf/). Following a short discussion, participants used Mentimeter to vote anonymously on whether the check should be included in the tool. Because each meeting was held in duplicate, there was scope for disagreement between groups. Where this occurred, participants from both meetings were invited to anonymously present arguments for and against the check using a collaborative online tool (Google Jamboard). All participants were subsequently invited to vote again via an online survey, implemented in Qualtrics, and the consensus criterion was applied to determine inclusion.

The meetings were held in June and July 2024. Twenty-one checks were selected for inclusion on the basis of the consensus meetings and subsequent resolution of four disagreements (Supplementary Table 3). A draft tool was created including these 21 checks, which were reorganised and reworded in light of the consensus meeting discussions. One domain was renamed, one was dropped, and a new domain was introduced.

### User testing of the draft tool

In Stage 5, the draft tool was tested and feedback was gathered from testers. Individuals who appraise RCTs as part of their role were recruited. Individuals were supplied with the draft tool and a summary guidance document, were asked to use these to assess the trustworthiness of one or more RCTs, and to provide feedback via an online survey (available at https://osf.io/y2c5u ). Participants were supplied with a participant information sheet and were asked to provide written consent to take part before being supplied with a personalised survey link. Participants were asked whether they would be willing to discuss their feedback at a user workshop (slides available at https://osf.io/truwq ).

Survey responses were collected between October 2024 and February 2025. Supplementary Table 4 shows a summary of responses. 148 individuals were invited to provide feedback, and 75 gave written consent to participate. 40 individuals provided feedback via the survey. The median (IQR) number of trials assessed was 3 (1.5 to 4). The median (IQR) time to assess an RCT using the tool was 45 (27 to 74) minutes. 28 (70%) participants said they would choose to use the tool in future work, with 11 (28%) saying they did not know whether or not they would do so. One person said they would not. 37 (93%) said that they thought the judgements they reached using the tool were reasonable. Challenges with implementing several checks were highlighted.

The user feedback workshop was attended by 8 participants on 14th January 2025. Anonymised minutes from the meeting are shown in Supplementary Table 5. It was determined at the workshop that some challenges and user questions could be addressed by expanding the associated guidance document, and by providing a collection of examples for users of the tool. It was also determined that software would be useful to assist in the implementation of some of the checks, and that the guidance document should direct users to this software where available. The feedback was used to make minor changes to the tool (numbering for checks was added, some checks were reworded). In addition, a detailed version of the guidance document was produced, including examples of each check.

## The INSPECT-SR tool

**Figure 2:**
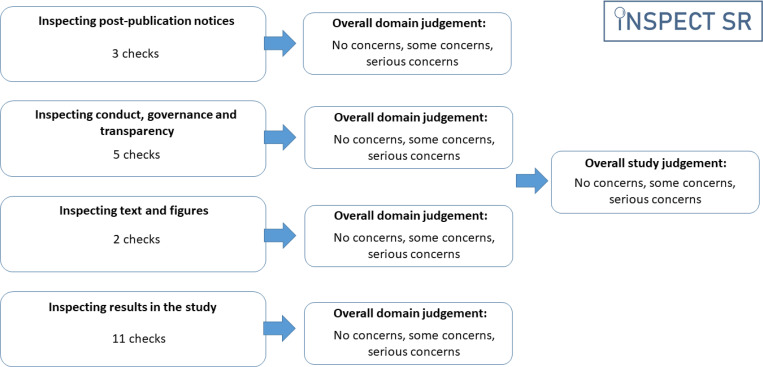
Structure of the INSPECT-SR tool

INSPECT-SR guides a reviewer through a series of up to 21 checks organised in four domains 1) *Inspecting post-publication notices* (3 checks), 2) *Inspecting conduct, governance, and transparency* (5 checks), 3) *Inspecting text and figures* (2 checks), 4) *Inspecting results in the study* (11 checks) (Table 1). An answer of “Yes” in response to a check indicates a potential problem. After completing the checks in a domain, the tool prompts the reviewer to make a judgement about the trustworthiness of an RCT in relation to that domain (“no concerns”, “some concerns”, or “serious concerns”), and to make an overall judgement about the trustworthiness of the study on the basis of the domain-level judgements (Figure 2). A detailed guidance document, including examples of each check, and an editable Microsoft Word template are provided at https://osf.io/b74wj/files/osfstorage.

## Using INSPECT-SR

Users should consult the detailed guidance document (https://osf.io/b74wj/files/osfstorage) before using INSPECT-SR. An overview of key points is provided here.

### Using check responses to arrive at an overall judgement

The guidance document includes instructions and examples for each check in the tool. The tool does not use a prescriptive algorithm to derive domain-level judgements from check responses. For example, we have not specified a threshold corresponding to a number of “Yes” responses to checks required to arrive at a domain-level judgement of “serious concerns”. Rather, the checks are intended to help the reviewer to reach a domain-level judgement about trustworthiness, and to articulate the rationale for the judgement. An answer of “Yes” to an individual check should not automatically trigger a judgement of “serious concerns”, although in some circumstances the problem highlighted by a check may provide a sufficient basis for doing so. For example, if the answer to check 1.1 *- Does the study have an associated retraction?* is “Yes” then this would usually be sufficient grounds to arrive at a domain-level judgement of “serious concerns”. In principle, a reviewer may decide that they have sufficient evidence to arrive at a domain-level judgement of “serious concerns” at any point during the assessment. If this happens, the reviewer may stop the assessment, assigning “serious concerns” to the study overall.

The overall study-level judgement should typically be at least as severe as the most severe domain-level judgement. This means that the study-level judgement should be at least “some concerns” if one or more domain-level judgements are “some concerns” and no domain-level judgement is “serious concerns”, and should be “serious concerns” if any domain-level judgement is “serious concerns”. A reviewer may also consider a study-level judgement of “serious concerns” to be appropriate on the basis of “some concerns” for several domains. In the event that “serious concerns” are identified, the reviewer should reflect on the rationale for the judgement to ensure that it is warranted. Regardless of the overall judgement, the reasons for the domain and study-level judgements should be clearly reported. The editable template (https://osf.io/b74wj/files/osfstorage ) includes space to add free-text comments.

The domains and checks in INSPECT-SR have been deliberately arranged to support an efficient assessment process, with relatively straightforward but definitive checks, such as checks for post-publication notices, appearing early in the list. However, reviewers may assess domains (or checks within domains) in a different order if they prefer to do so.

### Incorporating INSPECT-SR into the systematic review process

In the context of a systematic review, we recommend that INSPECT-SR is applied to assess trustworthiness of all eligible RCTs. We advise that INSPECT-SR should be used before risk of bias assessment and data extraction, to avoid wasting time assessing the risk of bias of, and extracting data from, problematic trials. RCTs with “serious concerns” should be excluded from the systematic review. RCTs with “some concerns” should be subjected to sensitivity analysis (for example, by comparing an analysis restricted to RCTs with “no concerns” to one that also includes RCTs with “some concerns”).

We recommend that INSPECT-SR be applied independently by two reviewers, who should then confer to reach consensus in relation to an assessment. It is likely to be beneficial to include at least one reviewer with content expertise as part of the assessment. While methodological expertise is also likely to be useful, use of the tool does not require advanced statistical knowledge. We recommend contacting study authors to attempt to resolve uncertainties. Templates for reporting trustworthiness concerns to journals are available from Cochrane (https://www.cochranelibrary.com/cdsr/editorial-policies/problematic-studies-implementation-guidance#7-1 ).

## Discussion

INSPECT-SR provides a rigorously developed, systematic and transparent method for identifying problematic RCTs in systematic reviews. It includes a series of checks that have been selected on the basis of empirical evidence and expert consensus. For a majority of reviewers, methods for the assessment of trustworthiness issues of this kind will be unfamiliar. This introduces important considerations for implementation. First, there is a need to have clear guidance to limit misapplication and misinterpretation of the checks contained in the tool. We have developed an accompanying guidance document, accessible at https://osf.io/b74wj/files/osfstorage, for this purpose. The guidance has been made available in an updatable format, as we anticipate that it will evolve continually in response to user feedback, evaluation of the tool, and developments in software and trustworthiness research. We will also add further training materials to this location.

A second consideration is that there will be a learning curve associated with the use of INSPECT-SR, and it may take longer for novice users to apply the tool. The median time per RCT was reported as 45 minutes in user testing, which is not unreasonable given that these were first-time users with limited guidance to facilitate their assessments. However, there was considerable variation in this assessment time. This appears to be explained by several factors. First, the guidance prompts users to stop the assessment if they judge there to be “serious concerns” at any point, meaning that some assessment times will be very short. Another factor is that some testers opted to review all material related to a RCT - for example, carefully checking results in supplementary materials for errors, or reviewing all versions of the clinical trial registration record. While this will increase the chance of identifying problems, it might not be practical for many reviewers. A pragmatic approach might, for example, involve testing a selection of results for errors, rather than every testable result in the paper. AI-based tools could be useful in this regard in the future, if developed to acceptable standards (21). The use of large language models (LLMs) to expedite systematic review processes is an area of active research (22, 23) and this includes work exploring use of LLMs to assist with INSPECT-SR [UKRI Metascience research grant (OPP569) awarded to Avenell].

Systematic reviewers and guideline developers have a responsibility to identify problematic studies because the trustworthiness of evidence synthesis depends on the evidence it includes. Recent examples highlight the issue. A National Institute for Health and Care Excellence (NICE) recommendation on fetal pillow (Cooper-Surgical) has been reversed following the retraction of an RCT of the device (24). It is possible that a trustworthiness assessment could have prevented inclusion of the trial in the recommendation ahead of its retraction, as it appears to contain statistical anomalies, including in the abstract. During the COVID-19 pandemic, systematic reviews for ivermectin suggested an impressive benefit of the drug for reducing mortality on the basis of potentially problematic trials (25), while subsequent high-quality evidence appears to show modest or no benefit (26, 27). Examples of potentially problematic trials threatening the integrity of systematic reviews can be found across a diverse range of clinical areas (3, 10, 28). The cost of doing nothing at all could be severe.

There have historically been no widely agreed standards of trustworthiness assessment (3), leading to ad-hoc, obscure, and potentially inequitable assessments. INSPECT-SR attempts to address this gap by providing standardised criteria for transparent trustworthiness assessment, which have the backing of a large, international, expert consensus. Systematic reviewers should share the methods that they use for identifying problematic studies, as well as the results of their investigations. INSPECT-SR will enable the equitable application of a systematic method and, by providing a structured format for transparently articulating concerns, will minimise duplication of effort to identify problematic studies. Nonetheless, ongoing evaluation of INSPECT-SR will be important to identify areas of misuse and misinterpretation, to identify training needs, and to identify examples of good practice to use as exemplars. Findings from this work will be used to improve the INSPECT-SR guidance document. In this regard, we would caution against attempts to assess “diagnostic accuracy” of INSPECT-SR, for example, by comparing judgements obtained using the tool to retraction status of articles, if that is based on the assumption that this represents an objective gold standard. In reality, whether or not an article is retracted is a subjective decision that is to some degree arbitrary, such that identification of trustworthiness concerns in a non-retracted article might not reflect a failure of the tool so much as a failure of pre or post-publication editorial processes (16).

While several other trustworthiness tools have been proposed, there are important differences with INSPECT-SR in terms of development, scope, content, and form. For example, recently published trustworthiness guidelines from a collaboration of OBGYN Editors include consideration of adherence to reporting standards and outcome reporting bias (29), which are not within the scope of INSPECT-SR. REAPPRAISED is another tool that includes items that are out of scope of INSPECT-SR, such as consideration of appropriateness of statistical methods and content relating to animal research (30). Items that were in scope in REAPPRAISED were considered for inclusion in INSPECT-SR (18), and many checks eventually included in INSPECT-SR have predecessors in the earlier tool. The INSPECT-SR guidance cautions against some of the checks included in other tools or published guidance, such as applying a penalty for trials that under-recruit.

Routine trustworthiness assessment may act as a deterrent to the production of problematic studies. Excluding problematic studies from systematic reviews has the potential to improve the scientific literature by reducing citations of problematic studies and their use by researchers and policy makers (31, 32). Nonetheless, an important question for developers of trustworthiness tools is whether the tools could be used by fraudsters to produce more convincing forgeries. We expect that many fabricators have produced and published fake research on the reasonable assumption that no one would notice, or even consider the possibility of, the fakery and, on balance, we expect that INSPECT-SR is likely to detect and deter many more fake RCTs than it creates. It is also unclear to what extent efforts to circumvent INSPECT-SR, either by manual fabrication or using LLMs, will be successful. Both of these areas should be a focus of future research.

INSPECT-SR has been developed primarily for assessment of health-related RCTs in a systematic review context. We encourage systematic review producers to adopt INSPECT-SR. We also encourage publishers to support adoption of INSPECT-SR by recommending it in instructions to authors of systematic reviews. Updates to the PRISMA (Preferred Reporting Items for Systematic reviews and Meta-Analyses) statement should include guidance on reporting methods and results of trustworthiness assessment (33). Funders and commissioners of systematic reviews should recognise the value of routine trustworthiness assessment, and should recognise that it may be necessary to increase resources and timelines to support this activity.

Future work will develop versions of the tool for other study designs, including non-randomised studies of interventions. Variations of INSPECT-SR may facilitate identification of problematic studies for some other responsible parties. For example, a version of the tool for use by journal editors in editorial assessment, INSPECT-JR, is planned. While systematic reviewers can draw attention to problematic studies and use INSPECT-SR to improve the trustworthiness of systematic reviews, they cannot prevent publication of problematic studies. As noted previously, everyone involved in the scientific publication pipeline – researchers, journals, publishers, and funders – needs to work together to reduce publication of problematic studies (34).

## Supplementary Material

Supplement 1

## Figures and Tables

**Table 1:** INSPECT-SR

Domain and check	Response
**Inspecting post-publication notices**	
1.1. Does the study have an associated retraction?	Yes, No, Unclear or Not applicable
1.2. Does the study have an associated expression of concern or other relevant post publication notice?	Yes, No, Unclear or Not applicable
1.3. Do other studies by the research team highlight causes for concern (associated retractions, expressions of concern, relevant post-publication notices?)	Yes, No, Unclear or Not applicable
**Overall domain judgement**	**No concerns, some concerns, serious concerns**
**Inspecting conduct, governance, and transparency**	
2.1. Are there concerns relating to ethical approval?	Yes, No, Unclear or Not applicable
2.2. Are there concerns relating to the timing or absence of study registration?	Yes, No, Unclear or Not applicable
2.3. Are there important inconsistencies between the publication and the registration documents?	Yes, No, Unclear or Not applicable
2.4. Is the recruitment of participants implausible?	Yes, No, Unclear or Not applicable
2.5. Are the reported methods implausible considering the reported resources?	Yes, No, Unclear or Not applicable
**Overall domain judgement**	**No concerns, some concerns, serious concerns**
**Inspecting text and publication details**	
3.1. Are there concerns relating to duplicated content, such as text or tables, or text that is incompatible with the study?	Yes, No, Unclear or Not applicable
3.2. Is there evidence of manipulation or duplication of figures?	Yes, No, Unclear or Not applicable
**Overall domain judgement**	**No concerns, some concerns, serious concerns**
**Inspecting results in the study**	
4.1. Are there any unexplained discrepancies between reported data and participant eligibility criteria?	Yes, No, Unclear or Not applicable
4.2. Are numbers of participants allocated to each group implausible given the allocation method?	Yes, No, Unclear or Not applicable
4.3. Are any baseline data implausible?	Yes, No, Unclear or Not applicable
4.4. Are there any discrepancies between results reported in figures, tables, and text?	Yes, No, Unclear or Not applicable
4.5. Are the numbers of participants lost to follow-up implausible?	Yes, No, Unclear or Not applicable
4.6. Are there any unexplained inconsistencies in the numbers of participants?	Yes, No, Unclear or Not applicable
4.7. Are any outcome data, including estimated treatment effects, implausible?	Yes, No, Unclear or Not applicable
4.8. Are the means and variances of integer data impossible?	Yes, No, Unclear or Not applicable
4.9. Are there errors in statistical results?	Yes, No, Unclear or Not applicable
4.10. Are any other contradictions implied by the data?	Yes, No, Unclear or Not applicable
4.11. Are there inconsistencies in descriptions of methods and results across publications describing the study?	Yes, No, Unclear or Not applicable
**Overall domain judgement**	**No concerns, some concerns, serious concerns**
**Overall study judgement**	**No concerns, some concerns, serious concerns**
