# Supplementary material for: INSPECT-SR: a tool for assessing trustworthiness of randomised controlled trials
Source: medRxiv. 2025 Sep 11:2025.09.03.25334905. Originally published 2025 Sep 5. Preprint. [Version 2] doi: 10.1101/2025.09.03.25334905 (PMC12424918; doi:10.1101/2025.09.03.25334905)
Supplement: Supplementary file 1 [file NIHPP2025.09.03.25334905v2-supplement-1.pdf]

medRxiv preprint doi: https://doi.org/10.1101/2025.09.03.25334905; this version posted September 11, 2025. The copyright holder for this preprint (which was not certified by peer review) is the author/funder, who has granted medRxiv a license to display the preprint in perpetuity.
It is made available under a CC-BY 4.0 International license .

Supplementary Tables:

		Round = 1	Round = 2
N		148	134
In which country do you primarily work?	Australia	24 (16.2%)	19 (14.3%)
	Austria	3 (2.0%)	3 (2.3%)
	Canada	6 (4.1%)	4 (3.0%)
	China	3 (2.0%)	7 (5.3%)
	Colombia	0 (0.0%)	1 (0.8%)
	Denmark	4 (2.7%)	4 (3.0%)
	Egypt	4 (2.7%)	4 (3.0%)
	France	7 (4.7%)	4 (3.0%)
	Germany	4 (2.7%)	5 (3.8%)
	Greece	1 (0.7%)	1 (0.8%)
	India	2 (1.4%)	1 (0.8%)
	Ireland	3 (2.0%)	3 (2.3%)
	Italy	1 (0.7%)	1 (0.8%)
	Malawi	1 (0.7%)	0 (0.0%)
	Malaysia	1 (0.7%)	1 (0.8%)
	Netherlands	3 (2.0%)	3 (2.3%)
	New Zealand	3 (2.0%)	3 (2.3%)
	Nigeria	1 (0.7%)	0 (0.0%)
	Poland	2 (1.4%)	1 (0.8%)
	Romania	1 (0.7%)	1 (0.8%)
	Spain	1 (0.7%)	1 (0.8%)
	Sweden	4 (2.7%)	5 (3.8%)
	Switzerland	1 (0.7%)	1 (0.8%)
	United Kingdom	58 (39.2%)	52 (39.1%)
	USA	8 (5.4%)	6 (4.5%)
	Viet Nam	2 (1.4%)	2 (1.5%)
Do you have expertise or experience in assessing potentially problematic studies, or other relevant expertise?	Yes	135 (91.2%)	123 (92.5%)
Have you assessed potentially problematic studies as a journal editor?	Yes	39 (28.9%)	36 (29.3%)
Have you assessed potentially problematic studies as a peer reviewer (prior to publication)?	Yes	75 (55.6%)	74 (60.2%)
Have you assessed potentially problematic studies as an independent researcher (post-publication)?	Yes	104 (77.0%)	97 (78.9%)
Have you assessed potentially problematic studies at the request of a journal or publisher?	Yes	30 (22.2%)	28 (22.8%)
Have you assessed potentially problematic studies as a research integrity professional?	Yes	30 (22.2%)	25 (20.5%)
Have you assessed potentially problematic studies you have been involved in (e.g. possible misconduct by collaborators)	Yes	20 (14.8%)	16 (13.1%)
Have you assessed potentially problematic studies in any other capacity not listed?	Yes	34 (25.2%)	31 (25.4%)
Roughly how many potentially problematic studies have you assessed?	1 to 10	67 (49.6%)	56 (45.5%)
	11 to 20	16 (11.9%)	17 (13.8%)
	More than 20	45 (33.3%)	46 (37.4%)
	None	7 (5.2%)	4 (3.3%)
Do you have other relevant expertise?	Yes	80 (59.3%)	74 (60.2%)

Supplementary Table 1: Characteristics of Delphi participants

medRxiv preprint doi: https://doi.org/10.1101/2025.09.03.25334905; this version posted September 11, 2025. The copyright holder for this preprint (which was not certified by peer review) is the author/funder, who has granted medRxiv a license to display the preprint in perpetuity.
It is made available under a CC-BY 4.0 International license .

Check	Usefulness	Feasibility	% scoring 7+ usefulness
Are there typographical errors?	4(3 to 5)	9(8 to 9)	12.9
Has the study been retracted or does it have an expression of concern, a relevant post-publication amendment, a critical Retraction Watch or PubPeer comment or has been previously excluded from a systematic review?	9(9 to 9)	9(8 to 9)	100
Is there evidence of copied work, such as duplicated or partially duplicated tables or figures?	9(8 to 9)	5(4 to 6)	97
Is there evidence of text reuse (cutting and pasting text between papers), including text that is inconsistent with the study?	8(7 to 8)	6(5 to 7)	81.8
Is there evidence of automatically-generated text?	7(5 to 8)	5(4 to 6)	55.8
Was the study published in a predatory journal?	7(5 to 8)	7(6 to 8)	61.4
Is there evidence of manipulation or duplication of images?	9(8 to 9)	5(4 to 6)	96.2
Are important features missing from the paper?	7(5 to 8)	8(7 to 8)	54.7
Was the time between submission to acceptance reasonable?	6(5 to 7)	9(8 to 9)	39.5
Is contact information for the author team present and plausible?	7.5(6 to 9)	9(8 to 9)	72.7
Are any baseline data implausible with respect to magnitude, frequency, or variance?	9(8 to 9)	7(6 to 8)	96.9
Is the number of participants lost to follow-up compatible with the population, setting, and timeline?	8(7 to 8)	7(6 to 8)	93.1
Are subgroup means compatible with those for the whole cohort?	7(7 to 8)	7(6 to 8)	84.7
Are the reported summary data compatible with the reported range?	8(7 to 9)	8(7 to 9)	93.8
Are correct units reported?	7(6 to 8)	8(8 to 9)	59.2
Are calculations of proportions and percentages correct?	7(6 to 8)	9(8 to 9)	68.5
Are numbers of participants correct and consistent throughout the publication?	8(7 to 9)	9(8 to 9)	82.3
Are there any discrepancies between data reported in figures, tables and text?	8(7 to 9)	9(8 to 9)	82.4
Are any outcome data, including estimated treatment effects, implausible?	8(8 to 9)	7(7 to 8)	92.4
Are statistical tests correct?	8(8 to 9)	6(5 to 7)	90.8
Are differences in variances in baseline variables between randomised groups plausible?	7(7 to 8)	6(5 to 7)	77.9
Are any of the baseline data excessively **similar** between randomized groups?	8(7 to 8)	7(6 to 8)	78.3
Are any of the baseline data excessively **different** between randomised groups?	7(7 to 8)	7(6 to 8)	75.4
Are the summary outcome data identical or nearly identical across study groups?	7(6 to 8)	8(7 to 9)	73.2
Are there any discrepancies between the values for percentage and absolute change?	7(6 to 8)	7(7 to 8)	66.7
Are there any discrepancies between reported data and participant inclusion criteria?	7(7 to 8)	8(7 to 9)	81.6
Are the variances in biological variables surprisingly consistent over time?	7(7 to 8)	7(6 to 8)	83.8
Are results internally consistent?	8.5(8 to 9)	7(6 to 8)	95.4
Are coefficients of variation unusually similar when calculated across variables reported in the paper?	7(6 to 7)	7(5 to 7)	63.2
Is the amount of missing data plausible?	7(7 to 8)	7(6 to 8)	76.6
Are the results substantially divergent from the results of multiple other studies, e.g. in a meta-analysis?	7(6 to 8)	8(7 to 9)	70.2
Are terminal digits compatible with a genuine measurement process?	7(6 to 8)	6(5 to 7)	64.1
Are the means and variances of integer data impossible?	9(8 to 9)	7(6 to 8)	94.9
Is there heterogeneity across studies in degree of imbalance in baseline characteristics (in meta-analysis)	6(5 to 7)	6(5 to 7)	39.4

medRxiv preprint doi: https://doi.org/10.1101/2025.09.03.25334905; this version posted September 11, 2025. The copyright holder for this preprint (which was not certified by peer review) is the author/funder, who has granted medRxiv a license to display the preprint in perpetuity.
It is made available under a CC-BY 4.0 International license .

Are integer data simulated from reported summary statistics plausible?	7(6 to 8)	5(5 to 7)	67.8
Are the numbers of participants allocated to each group plausible given the allocation method?	8(7 to 9)	8(7 to 9)	89.1
Is the grant funding number identical to the number in unrelated studies?	8(7 to 9)	8(6 to 9)	77.3
Is a funding source reported?	6(5 to 7)	9(9 to 9)	40.9
Is the volume of work reported by the research group plausible, including that indicated by concurrent studies from the same group?	7(6 to 7)	6(5 to 7)	56.6
Is the reported staffing adequate for the study conduct as reported?	6(5 to 7)	5(4 to 7)	32.5
Is the recruitment and follow-up of participants plausible?	7(7 to 8)	7(6 to 8)	80.6
Is the interval between study completion and manuscript submission plausible?	7(6 to 8)	8(6 to 9)	67.7
Is there evidence that the work has been approved by a specific, recognized ethics committee?	9(8 to 9)	9(8 to 9)	92.2
Are there any concerns about unethical practice?	8(7 to 9)	7(6 to 7)	82.9
Could the study plausibly be completed as described?	8(7 to 9)	7(5 to 7)	89
Are the locations where the research took place specified, and is this information plausible?	7(6 to 7)	7(5.5 to 8)	59.7
Do the authors agree to share individual participant data, or are the data publically available?	8(7 to 9)	9(7 to 9)	76.9
Does the trial registration number refer to other studies?	9(8 to 9)	9(8 to 9)	93
Has the study been prospectively registered?	9(7 to 9)	9(9 to 9)	82.2
Are details such as dates and study methods in the publication consistent with those in the registration documents?	8(7 to 9)	9(8 to 9)	86.2
Do authors cooperate with and provide satisfactory responses to requests for information?	8(7 to 9)	8(6 to 9)	82.3
Is the procedure of the study aligned with local legislations?	7(5 to 7)	4(3 to 5)	50.8
In which country was the study conducted?	5(3 to 6)	9(9 to 9)	20.2
Are withdrawal and loss to follow-up rates in multiple trials by the same authors plausible?	7(7 to 8)	5(4 to 7)	76.4
Are contributorship statements present and complete?	5(5 to 6)	9(8 to 9)	23.4
Have the data been published elsewhere by the research team in an illegitimate fashion?	9(8 to 9)	6(5 to 7)	90.6
Are duplicate-reported **results** consistent between publications?	8(7 to 9)	7(5 to 8)	88.5
Are relevant **methods** consistent between publications?	7(7 to 9)	7(5 to 8)	77.8
Is any duplicate reporting acknowledged or explained?	7(5 to 8)	7.5(6 to 9)	58.3
Does the statistics methods section use generic language, suggesting lack of expert statistical input?	6(5 to 7)	7(6 to 7)	31.2
Are terminal digits from multiple studies by the same authors compatible with a genuine measurement process?	7(6 to 8)	5(4 to 6)	57.3
Does consideration of other studies from members of the research team highlight causes for concern (including expressions of concern, relevant post-publication amendment, or critical retraction)?	8(8 to 9)	7(7 to 8)	95.3
Are the results in multiple studies from the same author implausibly similar?	9(8 to 9)	6(5 to 7)	94.5
Do all authors meet criteria for authorship?	5(4 to 6)	5(3 to 6)	15.3
Is authorship of related papers consistent?	5(4 to 6)	6(4 to 7)	14.8
Are the authors on staff of institutions they list?	6(5 to 8)	6(5 to 7)	44.1
Do any authors have a professorial title but no other publications on PubMed?	6(5 to 7)	7(6 to 8)	37
Can co-authors attest to the reliability of the paper?	7(6 to 8)	5(4 to 6)	63.3
Given the nature of the study, does the author list make sense?	6(5 to 7)	6(5 to 7)	48
Are results robust to or explainable by recoding of outcomes?	7(6 to 8)	6(5 to 8)	61.2
Are results robust to or explainable by redaction of outcomes?	7(5 to 8)	6(5 to 8)	54.1

Supplementary Table 2: Results of the Delphi survey. Median (IQR) shown for usefulness and feasibility scores.

medRxiv preprint doi: https://doi.org/10.1101/2025.09.03.25334905; this version posted September 11, 2025. The copyright holder for this preprint (which was not certified by peer review) is the author/funder, who has granted medRxiv a license to display the preprint in perpetuity.
It is made available under a CC-BY 4.0 International license .

Check	Meeting 1 vote to include	Meeting 2 vote to include	Consensus vote (if applicable)	Discussion points	Inclusion in tool
Inspecting conduct, governance, and transparency					
Is the recruitment and follow-up of participants plausible?	9/9	7/7	NA	Recruitment and follow-up should be distinguished as separate checks.	2.4. Is the recruitment of participants implausible?<br><br>4.5. Are the numbers of participants lost to follow-up implausible?
Is there evidence that the work has been approved by a specific, recognized committee?	9/9	8/8	NA	Should check certification of committee. Should check uniqueness of approval number and name.	2.1. Are there concerns relating to ethical approval?
Are there any concerns about unethical practice?	7/9	1/8	8/16	Could be sign of fake study – authors have not thought through what would be acceptable. But quite vague, and potentially redundant given check of approval.	No (covered by 2.1 above)
Could the study plausibly be completed as described?	7/8	8/8	NA	Change wording to "conducted". Include consideration of whether funding was sufficient.	2.5. Are the reported methods implausible considering the reported resources?
Does the trial registration number refer to other studies?	3/9	3/8	NA	Can be covered by another check comparing publication details to registration documents	No (covered by 2.3 below)
Has the study been prospectively registered?	9/9	8/8	NA	Easy to do, so no excuse not to do it. Registering shortly after start of study might not be a cause for concern, so "prospective" might not be appropriate choice of wording.	2.2. Are there concerns relating to the timing or absence of study registration?
Are details such as dates and study methods in the publication consistent with those in the registration documents?	8/9	8/8	NA	Could potentially be expanded to include comparisons with other documents (protocol, other publications)	2.3. Are there important inconsistencies between the publication and the registration documents?<br><br>Comparisons with other publications covered by 4.11
Do authors cooperate with requests for information?	8/9	1/8	9/16	May be useful flag, but need to include "appropriately". Guidance will tell reviewers to contact authors to resolve issues; if not resolved there will be a mark against the study for the appropriate check. So no need to penalize twice.	No. Guidance states reviewers should contact authors to resolve discrepancies.
Inspecting results in the paper					
Are any baseline data implausible with respect to magnitude, frequency, or variance?	14/14	9/9	NA	Could include assessment of baseline p-value distribution (that check did not meet consensus threshold in Delphi survey).	4.3. Are any baseline data implausible?<br><br>Baseline p-value assessment discussed in relation to this check in guidance, but not routinely recommended.
Is the number of participant withdrawals compatible with the disease, age and timeline?	12/13	8/8	NA	"Withdrawals" unclear. We mean loss to follow-up for any reason.	4.5. Are the numbers of participants lost to follow-up implausible?
Are numbers of participants correct and consistent throughout the publication?	11/12	9/9	NA	Participant numbers might change due to loss to follow-up or withdrawal. So need to make clear only interested in unexplained inconsistencies.	4.6. Are there any unexplained inconsistencies in the numbers of participants?
Are any outcome data, including estimated treatment effects, implausible?	14/14	9/9	NA	Considered to be straightforward.	4.7. Are any outcome data, including estimated treatment effects, implausible?

medRxiv preprint doi: https://doi.org/10.1101/2025.09.03.25334905; this version posted September 11, 2025. The copyright holder for this preprint (which was not certified by peer review) is the author/funder, who has granted medRxiv a license to display the preprint in perpetuity.
It is made available under a CC-BY 4.0 International license .

Are statistical test results correct?	12/14	8/8	NA	Need to consider explanations (e.g. analysis might have been adjusted for covariates.) P-value might be very different when reproduced from rounded summary data. Might be more suspicious if all results can be reproduced from rounded summary data.	4.9. Are there errors in statistical results?<br><br>Guidance discusses concerns due to ability to reproduce results using summary data.
Are there any discrepancies between reported data and participant inclusion criteria?	11/14	9/9	NA		4.1. Are there any unexplained discrepancies between reported data and participant eligibility criteria?
Are the variances in biological variables surprisingly consistent over time?	1/14	4/9	NA	Difficult to assess. Could potentially be considered as part of plausibility of outcome data.	No.
Are the means and variances of integer data impossible?	13/14	5/5	NA		4.8. Are the means and variances of integer data impossible?
Are the numbers of participants allocated to each group plausible given the allocation method?	12/14	5/8	14/17	May pick up contradictions. More useful for considering all studies from one author than a single study. Don't want to overlap with risk of bias tools. Could potentially be assessed as part of plausibility of baseline characteristics?	4.2. Are numbers of participants allocated to each group implausible given the allocation method?
Are any contradictions implied by the results?	Participants voted on whether to include the next four checks as sub-checks here.				
Are subgroup means incompatible with those for the whole cohort?	13/14	7/8	NA	Considered useful, but might not frequently be testable	Included in 4.10. Are any other contradictions implied by the data?
Are the reported summary data compatible with the reported range?	14/14	5/6	NA		Included in 4.10. Are any other contradictions implied by the data?
Are there any discrepancies between data reported in figures, tables and text?	10/13	9/9	NA	Sufficiently important to be a standalone check	4.4. Are there any discrepancies between results reported in figures, tables, and text?
Are results internally consistent?	9/13	0/8	NA	Considered unclear, even after considering examples. Would need a wording change to avoid misuse (examples of contradictory results could be indicated under the more general check)	Contradictions can be highlighted in 4.10.
Inspecting text and publication details					
Has the study been retracted or does it have an expression of concern, a relevant post-publication amendment, a critical Retraction Watch or PubPeer comment or has been previously excluded from a systematic review?	9/9	6/7	NA	Spirit of the check correct but it bundles together too many distinct things. Retractions and EoC should be treated separately. Mixed views on role of PubPeer (it is possible a PubPeer comment could have no merit). Decided to include PubPeer in guidance document – reviewer might consider PubPeer comments to help them make an assessment, but presence of a comment should not automatically trigger concerns.	1.1. Does the study have an associated retraction?<br>1.2. Does the study have an associated expression of concern or other relevant post publication notice?
Is there evidence of copied work, such as duplicated or partially duplicated tables?	1/8	5/6	6/12	Agreed that this would be useful, but there were concerns about feasibility at current time. This could be included in a check concerning plagiarism to allow	Included in 3.1

medRxiv preprint doi: https://doi.org/10.1101/2025.09.03.25334905; this version posted September 11, 2025. The copyright holder for this preprint (which was not certified by peer review) is the author/funder, who has granted medRxiv a license to display the preprint in perpetuity.
It is made available under a CC-BY 4.0 International license .

				instances of duplicated tables to be highlighted if noticed. Software solutions might improve feasibility.	
Is there evidence of text reuse (cutting and pasting text between papers), including text that is inconsistent with the study?	8/8	6/7	NA	Focus should be on inconsistent text (because plagiarism detection software not universally available). Duplicated text can be highlighted with this check if detected.	3.1. Are there concerns relating to duplicated content, such as text or tables, or text that is incompatible with the study?
Is there evidence of manipulation or duplication of images?	7/7	5/7	NA	Unclear whether software is useful for the types of figures found in RCT publications. Manual checks useful in the absence of software.	3.2. Is there evidence of manipulation or duplication of figures?
Inspecting the research team and their other work					
Have the data been published elsewhere by the research team in an illegitimate fashion?	1/8	4/12	NA	Not relevant to trustworthiness of the RCT.	No
Are duplicate-reported data consistent between publications?	8/8	8/9	NA	Consistency of methods is just as important as consistency of results, so should be added.	4.11. Are there inconsistencies in descriptions of methods and results across publications describing the study?
Does consideration of other studies from members of the research team highlight causes for concern (including expressions of concern, relevant post-publication amendment, or critical retraction)?	8/8	11/11	NA	Discussion about whether track record of the author team should be used to assess the index RCT.	1.3. Do other studies by the research team highlight causes for concern (associated retractions, expressions of concern, relevant post-publication notices?)
Are the results in multiple studies from the same author implausibly similar?	1/8	3/12	NA	Not feasible to consider all studies from the author team.	No

Supplementary Table 3: Results of consensus meetings

medRxiv preprint doi: https://doi.org/10.1101/2025.09.03.25334905; this version posted September 11, 2025. The copyright holder for this preprint (which was not certified by peer review) is the author/funder, who has granted medRxiv a license to display the preprint in perpetuity.
It is made available under a CC-BY 4.0 International license .

Survey response variable	Summary
**In what capacity have you appraised randomised controlled trials as part of your professional role? Please select all that apply**	
Health researcher	17 (41%)
Journal editor	12 (29%)
Peer reviewer	18 (44%)
Research integrity professional	4 (10%)
Systematic reviewer	34 (83%)
Other	7 (17%)
**In which country do you primarily work?**	
Australia	4 (10%)
Austria	1 (2%)
Canada	2 (5%)
China	2 (5%)
Colombia	1 (2%)
Denmark	2 (5%)
Egypt	1 (2%)
Finland	1 (2%)
France	2 (5%)
Germany	1 (2%)
Greece	2 (5%)
Ireland	1 (2%)
Italy	1 (2%)
Romania	1 (2%)
Sweden	4 (10%)
United Kingdom of Great Britain and Northern Ireland	11 (28%)
United States of America	3 (8%)
**How did you use the draft trustworthiness tool?**	
Some other way (To assess some randomized controlled trials included in a systematic review conducted by someone else and to assess one randomized controlled trial outside a systematic review context)	1 (2%)
To assess one or more randomised controlled trials outside of a systematic review context.	7 (18%)
To assess randomised controlled trials during the conduct of a new systematic review	17 (42%)
To assess randomised controlled trials in the update of an existing systematic review	2 (5%)
To assess randomised controlled trials included in a systematic review conducted by someone else.	3 (8%)
To assess randomised controlled trials included in a systematic review I was previously involved in.	10 (25%)
**How many trials did you assess using the draft INSPECT-SR tool? (median, IQR, range)**	3<br>1.5 to 4<br>1 to 19<br>1 no response
**Did you assess all of the trials that would have been eligible for inclusion in a systematic review?**	
No	26 (63%)
Not applicable – no systematic review	4 (10%)
Yes	10 (24%)
**Would you consider yourself to have topic expertise in relation to the trials you assessed?**	
I have a high level of topic expertise	12 (30%)
I have some topic expertise	17 (42%)
No	11 (28%)
**Approximate time taken to assess a trial (calculated from total time/ total number assessed, median, IQR, range)**	45, 27 to 74, 13 to 210
**What is your opinion about the time required to apply the draft tool?**	
It takes far too long to apply	3 (8%)
It takes somewhat too long to apply	16 (40%)
The time it takes to apply is reasonable	21 (52%)
**What is your opinion about the difficulty of applying the draft tool?**	
The difficulty is far too high	1 (2%)
The difficulty is somewhat too high	14 (35%)
The difficulty is reasonable	25 (62%)
**If applicable, would you choose to use it in future work?**	
I don’t know whether I would choose to use it in future work.	11 (28%)
No, it is unlikely that I would choose to use it in future work	1 (2%)
Yes, I would choose to use it in future work.	28 (70%)
**Do you think the judgements you arrived at using the draft tool were reasonable?**	
I don’t know	2 (5%)
No	1 (2%)
Yes	37 (92%)
**Did you reach a judgement of “serious concerns” for any of the trials you assessed?**	
No	21 (52%)
Yes	19 (48%)

Supplementary Table 4: Summary of quantitative responses to user testing survey

medRxiv preprint doi: https://doi.org/10.1101/2025.09.03.25334905; this version posted September 11, 2025. The copyright holder for this preprint (which was not certified by peer review) is the author/funder, who has granted medRxiv a license to display the preprint in perpetuity.
It is made available under a CC-BY 4.0 International license .

Topic	Summary of discussion
Structure of the tool	Liked how it was similar to ROBIS, felt familiar, not overwhelming<br><br>(People generally happy with this)
Approach to arriving at domain-level judgements	Even if 'some concerns' across all checks, wouldn't have moved domain to judgement to 'severe concerns'<br>+1 agree<br><br>Needs to be done by two people, preferably independently – people must have domain and stats proficiency (between them)<br><br>Prefer not algorithm driven (1 person)<br><br>Full guidance doc will help a lot<br><br>If not able to assess (due to lack of knowledge), what to use – 'unclear'? JW confirmed 'unclear' to be used that way<br><br>More difficult than risk of bias, more reliant on individual knowledge/communication with review authors<br><br>When systematic reviews are published would these INSPECT results be included? – JW: yes, articulation of judgement should be clear<br><br>JW: Should 'no information' be a domain level option? [e.g. if all checks in the domain unclear]<br>+1 against this<br>+1 for this<br><br>Need guidance on contacting authors to be consistent with Cochrane policy
Approach to arriving at an overall study-level judgement	JW: Should no prospective registration warrant 'serious concerns'?<br>+3 for no (expectations vary by region too greatly)<br>1 felt legality should be cut-off for expectation of registration<br><br>No other issues here
Time to complete	I don't see how it could be shorter, thinks 60-75 minutes wouldn't be too long, given the stakes. Was easier with studies I was familiar with<br><br>"Are there errors in statistical results" can be extremely time consuming. Resources to help people with that would be v.useful.<br><br>GRIM/GRIMMER v.useful and didn't take long<br><br>Papers that took longest were those that had 'some' concerns over multiple domains, required more in-depth investigation to determine final judgement.<br><br>Maybe these can be done as you're going along (i.e. you'll be reading the paper anyway as part of the systematic review, just do it then).<br><br>Order checks by severity (those most likely to find problems)? JW: We've had a go at doing that<br><br>JW: can potentially save time doing ROB if do this first
Difficulty	automation: awarded grant to help automate some of these checks

medRxiv preprint doi: https://doi.org/10.1101/2025.09.03.25334905; this version posted September 11, 2025. The copyright holder for this preprint (which was not certified by peer review) is the author/funder, who has granted medRxiv a license to display the preprint in perpetuity.
It is made available under a CC-BY 4.0 International license .

	Cochrane should have some central plagiarism check, should maybe be up to them<br><br>Duplicated text domain – if no information [no plagiarism software, no figures] the domain doesn't contribute anything (and therefore the weights of the other domains increase)<br><br>Plausibility (of results) checks – a sensitive subject, worried any area where mechanism of action is unknown may be targeted by reviewer with nefarious motivation (complementary med). Maybe get Cochrane Complementary to have a look<br>Plausibility is not just effect or not, about magnitude also. Reiterated point that having 2nd rater will help with this issue<br><br>Plausibility of other things (recruitment, etc), were helpful/uncontroversial. Guidance on this can make a big difference to ability to judge these checks<br><br>JW: Okay to update checks/ROB in light of meta-analysis? (i.e. noticing substantial difference in effect size in forest plot)<br>+2 for yes<br>+1 for no – should be fresh process every time, can't be any post-hoc retrospective work<br>Maybe a technical/reporting issue – what do you report, how to ensure transparency if you do go back and change assessment?
Any particularly challenging domains or checks?	JW: Open Q for 'other' concerns?<br>Already some free text boxes that can serve this purpose [JW: people put rubbish into 'other' boxes, and we don't want people using checks we don't endorse].<br><br>JW: reasonable to allow people to do their own order of domains?<br>First check must come first, otherwise – probably doesn't matter<br><br>Identification of 'critical' domain/checks may be option (as in AMSTART/ROBIS)
Overall impression, suggestions	JW: is scope of tool clear?<br>+1 for yes<br><br>Is application of tool going to be web-form or downloadable word doc?<br>JW: specifics of implementation to be worked out
Other	Definition of problematic studies (from Cochrane) includes unpublished study. Perhaps INSPECT should also? [JW: think it could be used, probably some domains unclear]<br><br>Should be more pointed mention of pubpeer plugin.<br><br>Problematic paper screener website? (for tortured phrases)

Supplementary Table 5: Anonymised minutes from user testing workshop
